# Pyroptosis and Its Regulation in Diabetic Cardiomyopathy

**DOI:** 10.3389/fphys.2021.791848

**Published:** 2022-01-25

**Authors:** Yafang Lu, Yaqiong Lu, Jun Meng, Zuo Wang

**Affiliations:** ^1^Institute of Cardiovascular Disease, Key Laboratory for Arteriosclerology of Hunan Province, Hunan International Scientific and Technological Cooperation Base of Arteriosclerotic Disease, Hengyang Medical College, University of South China, Hengyang, China; ^2^Functional Department, The First Affiliated Hospital, University of South China, Hengyang, China

**Keywords:** diabetic cardiomyopathy, pyroptosis, NLRP3 inflammasome, caspase-1, regulation

## Abstract

Diabetic cardiomyopathy (DbCM) is a prevalent disease, characterized by contractile dysfunction and left ventricular hypertrophy. Patients with DbCM have high morbidity and mortality worldwide. Recent studies have identified that pyroptosis, a kind of cell death, could be induced by hyperglycemia involved in the formation of DbCM. This review summarizes the regulatory mechanisms of pyroptosis in DbCM, including NOD-like receptor3, AIM2 inflammasome, long non-coding RNAs, microRNAs, circular RNA, autophagy, and some drugs.

## Introduction

Diabetes mellitus (DM) potentially causes increased risks of heart failure in the absence of the traditional impetus to heart failure such as hypertension and coronary heart disease, including, diabetic cardiomyopathy (DbCM) (Murtaza et al., 2019). In 1972, Rubler and his colleagues first described DbCM from the postmortem pathological findings of four diabetic patients who died of heart failure without evidence of hypertension and coronary artery disease (Jia et al., 2018b). In general, DbCM has two stages, namely, early stage featured by left ventricular hypertrophy and diastolic dysfunction and later stage featured by cardiac fibrosis and impaired systolic function (Paolillo et al., 2019). Multiple hallmarks have been reviewed to contribute to DbCM, including hyperglycemia, insulin resistance, lipid peroxidation, increased oxidative stress, mitochondrial dysfunction, cardiomyocyte calcium handling, endothelial dysfunction (Jia et al., 2018a), and cell death (Cai and Kang, 2003).

Based on the morphological features, cell death can be clearly classified into four types, namely, necrosis, autophagy, entosis, and apoptosis (Chen et al., 2020). Necrosis, an energy-independent and uncontrolled type of cell death, can be induced by some external injury such as inflammation and hypoxia (D’Arcy, 2019). Autophagy is a catabolic and regulated process associated with the formation of the autophagosome to engulf the cytoplasmic content. Entosis demonstrates “cell-in-cell” cytological features through lysosomal degradation (Martins et al., 2017). Apoptosis is featured by characteristic morphological changes such as cell shrinkage, pyknosis, and karyorrhexis. Some studies have shown that DbCM involves pyroptosis, which is a proinflammatory apoptotic process that differs from classic apoptosis (Li et al., 2014; Yang et al., 2018b; D’Arcy, 2019).

## Pathogenesis of Diabetic Cardiomyopathy

**FIGURE 1:**
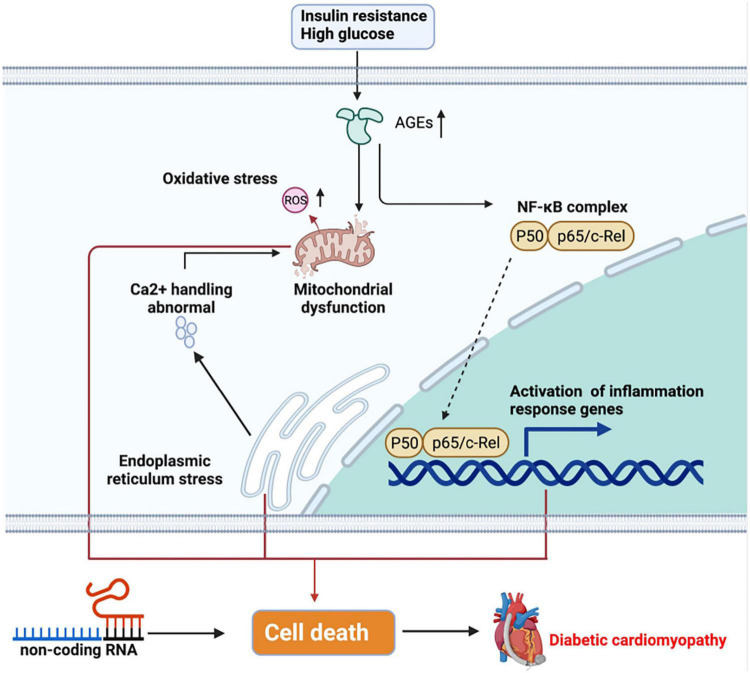
Pathogenesis of diabetic cardiomyopathy (DbCM). Insulin resistance and high glucose trigger cell metabolism disorder, further causing inflammation and cell death, which finally results in heart failure. ER, endoplasmic reticulum; AGEs, advanced glycated end-products; ROS, reactive oxygen species; NF-κB, nuclear factor-κB.

Of note, inflammation is involved in the pathogenesis of DbCM. Diabetes is a proinflammatory process, and several cytokines including interleukin-1β (IL-1β), IL-18, and tumor necrosis factor-alpha (TNF-α) are highly expressed in DbCM (Bugger and Abel, 2014). Insulin resistance and hyperglycemia in the heart have been found to be associated with the pathogenesis of DbCM. In physiological states, insulin regulates the myocardial metabolism, glucose transport, glycogen synthesis, and lipid metabolism. In a diabetic heart, the decreases in glucose transporter 4 and its abnormal translocation caused by insulin resistance limit the glucose uptake (Xia and Song, 2020). In contrast, cluster of differentiation 36 is preferentially located to the sarcolemma, which increases fatty acid oxidation in the diabetic heart. The changes in metabolisms prompt the deposition of lipids and advanced glycation end-products (AGEs) (Jia et al., 2016). Excessive lipids contribute to the mitochondrial dysfunction by affecting the respiratory function and mitochondrial biogenesis. Increased AGEs are associated with elevated generation of reactive oxygen species (ROS), which instigates inflammasome and fibrosis. ACEs result in crosslinks in collagen molecules, hence promoting collagen to be impaired and causing increased fibrosis, followed by elevated myocardial stiffness (Bugger and Abel, 2014). Furthermore, AGEs induced by hyperglycemia can lead to protein misfolding, which increases cytosolic calcium (Ca^2+^) from the endoplasmic reticulum and then induces Ca^2+^ disturbance in the mitochondria (Fiorentino et al., 2013). In addition, AGEs participate in the activation of nuclear factor κB (NF-κB), which controls DNA transcription and the formation of proinflammatory cytokine. Insulin resistance and hyperglycemia result in stress to the endoplasmic reticulum, which implicates impaired Ca^2+^handling. Impaired Ca^2+^ reuptake by the sarcoplasmic reticulum increases the duration of the action potential and decreases the diastolic relaxation of cardiomyopathy (Jia et al., 2018a). These pathophysiological abnormalities together cause cardiomyocyte death and finally cardiac stiffness and heart failure. Obviously, myocardial death is a crucial element in DbCM. A study showed that the expression of apoptosis and necrosis markers is high in the heart tissue of patients with type 2 diabetic (Frustaci et al., 2000). Recently, increasing studies have indicated the roles of non-coding RNAs (ncRNAs) in the pathogenesis of DbCM, including cell death, oxidative stress, myocardial fibrosis, and inflammation. For example, miR-1 could induce apoptosis in DbCM, while miR-22 could regulate hyperglycemia-induced oxidative stress (Zhang et al., 2019; Figure 1).

## Pyroptosis

**FIGURE 2:**
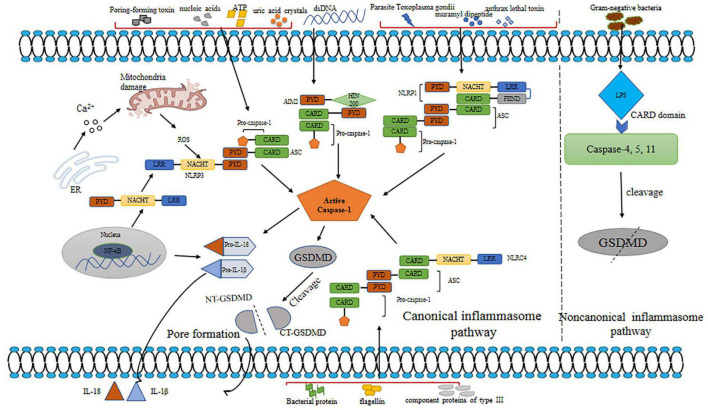
Mechanisms of pyroptosis. Pyroptosis is triggered by canonical and non-canonical inflammasome pathways. Various inflammasomes involved in canonical pathway are activated by different DAMPs and PAMPs. Canonical inflammasomes bind to ASC to activate caspsase-1. Caspase-1 plays a role in maturation of IL-1β and IL-18 and cleavage of GSDMD. N-GSDMD releases and forms a membrane pore, leading to the secretion of IL-1β and IL-18. In non-canonical pathway, LPS binds to human caspase-4/5 and mice caspase-11 to promote cleavage of GSDMD. dsDNA, double-stranded DNA; ROS, reactive oxygen species; AIM2, absent in melanoma 2; ASC, apoptosis-associated speck-like protein containing a caspase recruitment domain; NLRP3, NOD-like receptor; PYD, pyrin domain; LRR, leucine-rich repeat; NACHT, nucleotide binding and oligomerization; HIN-200, the interferon (IFN)-inducible p200-protein; GSDMD, gasdermin D; NT-GSDMD, N-terminal gasdermin D; CT-GSDMD, C-terminal gasdermin D; LPS; lipopolysaccharide; DAMPs, danger-associated molecular patterns; PAMPs, pathogen-associated molecular patterns; IL-18, interleukin-18; IL-1β, interleukin-1β.

The term pyroptosis comes from the Greek word “pyro,” meaning fever or fire, and ptosis, meaning falling. It depicts proinflammatory programmed cell death (Cookson and Brennan, 2001). According to the definition, pyroptosis is a proinflammatory type of programmed cell death, depending on the enzymatic activity with the involvement of inflammatory proteases, which are a part of the caspase family (Vande Walle and Lamkanfi, 2016). Pyroptosis leads to DNA damage, less DNA laddering, and chromatin condensation with an intact nucleus. Compared with apoptotic cells, pyroptotic cells are positive in TUNEL staining. Although DNA damage relies on caspase-activated DNase (CAD) and restricted CAD inhibitor during apoptosis, CAD is not needed during pyroptosis (Jorgensen and Miao, 2015; Fang et al., 2020). Furthermore, pyroptosis induces the formation of caspase-1-dependent membrane pores with 1.1–2.4 nm diameter, which causes swelling and osmotic lysis (Fink and Cookson, 2006). Pyroptosis responds to infectious diseases and protects host cells against microbial pathogens; however, caspase activation is harmful to the body. Traditionally named caspase-1-mediated cell death, pyrotosis involves human caspase-4 and caspase-5, mouse caspase-11 (Kayagaki et al., 2015), and caspase-3 (a hallmark of apoptosis) (Cao et al., 2017). These caspases cleave and activate some specific members of the gasdermin gene family, leading to the occurrence of pyroptosis. The molecular mechanisms of pyroptosis are shown in Figure 2.

Inflammasomes are multimolecular protein complexes that typically contain a sensor protein pattern-recognition receptor (PRR), a proinflammatory caspase, and an adaptor protein apoptosis-associated speck-like protein containing a caspase recruitment domain (CARD) (ASC) (Rathinam and Fitzgerald, 2016). The PRR family includes Toll-like receptors, absent in melanoma 2 (AIM2) and NOD-like receptors (NLRs) (Dolasia et al., 2018; Zhao et al., 2020), which recognize danger-associated molecular patterns (DAMPs) induced by endogenous pathogens or pathogen-associated molecular patterns (PAMPs) derived from invading pathogens (Man and Kanneganti, 2015; Fang et al., 2020). ASC connects the sensor protein and pro-caspase-1 after PRR recognizes DAMP/PAMP, further promoting pyroptosis. Previous studies have demonstrated that there are generally canonical and non-canonical inflammasome pathways triggering pyroptosis.

Canonical inflammasomes include NOD-like receptor 1 (NLPR1), NLRP3, NLRC4, and AIM2, triggering pyroptosis by activating caspase-1 (Aachoui et al., 2013). NLRP1, NLRP3, and AIM2 have a pyrin domain (PYD) (Wang B. et al., 2019), and NLRC4 contains an N-terminal (NT) CARD, which enables them to recruit pro-caspase-1. Various inflammasomes are activated by special DAMPs and PAMPs. NLPR1 activates muramyl dipeptide and anthrax lethal toxin in response to parasite *Toxoplasma gondii* (Franchi et al., 2009; Ewald et al., 2014). AIM2 is a member of the AIM2-like receptor family containing one or two HIN domains and NT PYD (Wang and Yin, 2017). The HIN domain contains two oligosaccharide folds that can recognize nucleic acids, while the PYD domain regulates the homotypic protein-protein interaction. In homeostasis, AIM2 senses self-DNA by conserved mechanisms, which have been evolved to degrade mislocalized DNA or separate self-DNA to the nucleus (Kawane et al., 2014). Once double-stranded DNA (dsDNA) is recognized by PYD, AIM2 recruits ASC, activating caspase-1 in turn (Wang and Yin, 2017; Wang B. et al., 2019). AIM2 detects cytoplasmic dsDNA, which could be derived from bacteria, viruses, or host (Wang and Yin, 2017). NLRC4 recognizes bacterial proteins including flagellin, a component protein of type III secretion system. NLRP3 senses wide-ranging stimuli, including endogenous and infectious DAMPs such as microbial components; pore-forming toxins; nucleic acids; endogenous molecules such as uric acid crystals and ATP; and common cellular distress molecules like mitochondrial dysfunction, ROS, Ca^2+^, and rupture of lysosomes (Malik and Kanneganti, 2017; Kelley et al., 2019). The AIM2 or NLR signaling domains (CARD or PYD) connect ASC *via* homotypic interactions, inducing the formation of the ASC focus, which further recruits procaspase-1 (Aachoui et al., 2013). Subsequently, procaspase-1 is self-cleaved into bioactive caspase-1 composed of two p10 and two p20 subunits, which promote the maturation of IL-1β and IL-18 and induces the cleavage of gasdermin D (GSDMD) to form the membrane pore (Fink et al., 2008; Boucher et al., 2018). The formation of membrane pores results in water influx, cell swelling, and lysis, in turn inducing pyroptotic cells to release inflammatory cytokines and DAMPs (Tsuchiya et al., 2019).

The non-canonical inflammasome pathway induces the activation of human caspase-4/5 and mouse caspase-11 (Broz and Dixit, 2016). As apical activators, caspase-4/5/11 directly detect lipopolysaccharide (LPS) in the host cytoplasm derived from Gram-negative bacteria by their CARD domains, causing the activation of GSDMD (Man et al., 2017). Furthermore, studies showed that caspase-4/5/11 cannot process IL-18 and IL-1β directly, but they induce GSDMD-mediated potassium efflux to promote the formation of the NLRP3 inflammasome and IL-1β activation (Ramirez et al., 2018; Frank and Vince, 2019). Wang et al. (2020) showed that caspase-4/11 autoprocess p10, which promotes the cleavage of GSDMD.

As the effector of pyroptosis, GSDMD was found to form pores in the membrane. Caspase-1/4/5/11 cleaves the linker sequence between the C-terminal (CT) and NT domains of GSDMD (Pandeya et al., 2019). GSDMD-CT is considered to bind to GSDMD-NT to restrict its activation (Liu et al., 2016). GSDMD-NT targets cardiolipin and phosphoinositide and oligomerizes to form pores with a diameter of 10–33 nm. Different from most pore-forming proteins, GSDMD lyses cell membranes from the cytosol (Ding et al., 2016; Man et al., 2017). GSDMD-NT also activates canonical NLRP3 inflammasome possibly by inducing K + efflux (Rühl and Broz, 2015).

Apoptosis is controlled by the caspase family and Bcl-2 protein family. Two pathways induce apoptosis, namely, extrinsic death pathway and intrinsic mitochondrial pathway. In the extrinsic pathway, caspase-8 is activated *via* Fas-associated death domain, which further activates caspase-3 after the combination of some substrates such as TNF-α and Fas-ligand-induced receptor trimerization (Sohns et al., 2010). In the intrinsic pathway, a number of proteins named the permeability transition pore complex form a pore in mitochondrial membranes, facilitating the release of mitochondrial proteins such as cytochrome C into the cytosol, which is involved in the formation of the apoptosome. Hereafter, the apoptosome activates procaspase-9 and caspase-3 (Chen et al., 2020).

Apoptosis leads to cell shrinkage, blebbing, and regular DNA fragmentation maintaining the membrane integrity, which protects normal surrounding cells from inflammatory response. GSDMD-dependent pyroptosis is characterized by integrated nuclear changes, cell swelling, lysis, and the release of cytokines, promoting inflammatory response. Moreover, during apoptosis, signal molecules activate proapoptotic proteins including Bad and Bid, which further repress the antiapoptotic protein Bcl family and facilitate Bax/Bak oligomerization, affecting the mitochondria (Sohns et al., 2010). Emerging studies found that GSDMD and gasdermin E (GSDME) permeabilize *via* the mitochondrial membrane and form pores in the mitochondrial membrane, releasing cytochrome C in pyroptosis (Rogers and Alnemri, 2019). Zheng et al. (2020) demonstrated that highly expressed BH3-only protein Bcl-2/adenovirus EIB 19-KDa-interacting protein 3 activates caspase-3, further inducing GSDME to facilitate pyroptosis in cardiomyocytes treated with doxorubicin. These results show that caspase-3 can regulate not only pyroptosis but also apoptosis in cardiomyocytes, and the generation of GSDME may be a sign to clarify these two kinds of cell death. Nevertheless, whether caspase-3 induces GSDME in cardiomyocytes treated with high glucose remains unknown.

## Pyroptosis in Diabetic Cardiomyopathy

Previous studies found the overexpression of NLRP3, caspase-1, and IL-1 in the heart of rats with diabetes (Zhou et al., 2018; Xie et al., 2020). Increasing pyroptosis is commonly observed in patients with diabetes. In addition, except hyperglycemia, high lipid, insulin resistance, and hyperinsulinemia all contribute to pyroptosis in DbCM *via* increasing ROS (Lee and Kim, 2017; Kar et al., 2019; Urdaneta Perez et al., 2020), which is seen as a significant activator inducing the NLRP3 inflammasome. Pyroptosis has been identified to occur in various heart cell types in DbCM including cardiomyocytes, endothelial cells (ECs), and cardiac fibroblasts (Qiu et al., 2017; Zhaolin et al., 2019; Chen et al., 2020). These various pyroptotic cells present similar and different features in DbCM.

Cardiomyocytes maintain the heart systolic and diastole function and help the heart pump blood to the body. ELAV-like protein 1 (ELAVL1) is a kind of RNA stabilizing protein that binds to AU-abundant elements in the 3′-untranslated region of different cytokines (Zhou et al., 2019). In high-glucose condition, high levels of caspase-1 and ELAVL1 were detected in cardiomyocytes. Furthermore, cells with ELAVL1 knockdown express lesser NLRP3 and caspase-1 than those with ELAVL1. ELAVL1 has been proved to modulate pyroptosis in cardiomyocytes (Jeyabal et al., 2016). Cardiomyocyte pyroptosis triggers cardiac fibroblasts to repair injury by releasing cytokines such as IL-1β, ultimately causing heart hypertrophy and systolic and diastolic dysfunction. In high-glucose condition, metabolic disorders are easily found in cardiomyocytes. Under hyperglycemia condition, increasing glycolysis supplies a source of ATP, which may disturb mitochondria by upregulating glucose-6-phospate (G6PD). G6PD produces NADPH, whose dehydrogenase triggers superoxide anion and NADPH oxidase to further damage the mitochondria. Mitochondrial dysfunction plays a pivotal role in the activation of the NLRP3 inflammasome. However, as the NLRP3 inflammasome also promotes apoptosis in cardiomyocytes (Zhang et al., 2018) and considerable evidence proves myocardial apoptosis in DbCM, it makes sense to distinguish apoptosis and pyroptosis.

Endothelial cells separate blood and tissue, which is a part of the inner layer of the blood vessel. ECs play a vital role in maintaining the vascular tone and structure. Moreover, as a natural anticoagulant tissue, ECs release several kinds of anticoagulant molecules such as heparin. Meanwhile, ECs participate in the immune system, secreting cytokines and swallowing bacteria and necrotic tissues. Previously, Chen H. et al., 2016 found that cadmium triggers NLRP3-mediated pyroptosis in ECs. This study indicated that high glucose induces the formation of NLRP3 inflammasome in coronary arterial ECs of mice. In addition, endothelial dysfunction was observed, and the deficiency of NLRP3 inflammation alleviated the injury of ECs (Chen Y. et al., 2016). This study illustrated that hyperglycemia-induced NLRP3 inflammasome is involved in the EC dysfunction. NLRP3 inflammasome-induced IL-1 targets ECs to induce various inflammatory activities such as increased expression of adhesion molecules. Moreover, ECs produce IL-1 to increase inflammation (Grebe et al., 2018; Bai et al., 2020), which further aggravates DbCM. Furthermore, high glucose triggers ECs to reduce the secretion of nitric oxide (NO), which is recognized as the most important vasodilator of ECs. In certain conditions, nitric oxide synthase (NOS) reduces L-arginine to L-citrulline and NO, which prevent inflammation and maintain vascular homeostasis. However, acute inflammatory cells and ECs are able to express arginase, which locally decomposes L-arginine to decrease the substrate of eNOS. Moreover, eNOS merely plays a role in producing NO in the presence of tetrahydrobiopterin (BH4) and heme. Otherwise, it works as NADPH oxidase in oxidative reaction (Cyr et al., 2020). BH4 deficiency is closely associated with inflammation in the retina. Thus, high-glucose-induced inflammatory process inhibits NO production of ECs by oxidizing BH4. Along with NO deficiency, more platelets adhere and gather in the blood vessel to promote thrombus, further aggravating heart failure.

Cardiac fibroblasts remodel the heart by promoting the excretion of collagen and other extracellular matrix components when the heart suffers damage and inflammation. However, if the damage is too large, too much collagen causes cardiac fibrosis, stiffness, and dysfunction. In DM, heart failure occurs partly because of the excessive excretion of the extracellular matrix from cardiac fibroblasts. Micro-RNA 21 was demonstrated to target dual specific phosphatase 8 to increase the production of collagen in the cardiac fibroblasts in high glucose (Liu et al., 2014). Moreover, high expression of NLRP3 and caspase-1 was found in cardiac fibrosis tissue of rats with diabetes. Another study indicated that miR-21-3p was expressed in high glucose. The inhibitor of miR-21-3p reduces caspase-1 in hyperglycemia, which regulates cardiac fibroblast pyroptosis. These findings show that hyperglycemia-induced pyroptosis mediated by miR-21-3p is associated with the dysfunction of cardiac fibroblast.

## Regulation of Pyroptosis in Diabetic Cardiomyopathy

Pyroptosis is involved in the pathogenesis of DbCM. Previous and emerging studies indicated that the regulations of pyroptosis in DbCM are complicated with the involvement of NLRP3 and AIM2 inflammasome, long non-coding RNAs (lncRNAs), micro RNA, circular RNA (circRNA), autophagy, and some drugs (Table 1).

**TABLE 1:** Drugs/genes, their targets, and function in diabetic cardiomyopathy (DbCM).

Drug/Gene	MiRNA	Target	*Vivo* trial	Target function	Expression
Metformin	/	AMPK	Yes	Reduce blood glucose	/
Empagliflozin	/	SGC	Yes	Reduce blood glucose	/
EUK-134	/	TXNIP NLRP3	No	Inhibit NLRP3 inflammasome	/
Skimmin	/	/	Yes	Enhance autophagy and inhibit pyroptosis	/
MNS	/	ATPase	Yes	Inhibit interaction of NEK7 and NLRP3	/
NSA	/	GSDMD	NO	Inhibit GSDMD cleavage and IL-1βrelease	/
BMP-7	/	/	Yes	Inhibit pyroptosis and improve cardiac remodeling	/
MALAT1	miR-141-3p		No	Promote pyroptosis	↓
GAS5	miR-34b-3p	AHR	Yes	Inhibit NLRP3-mediated pyroptosis	↓
Kcnq1ot1	miR-214-3P	/	Yes	Promote pyroptosis	↑
/	miR-30d	foxo3a ARC	Yes	Promote pyroptosis	↑
/	miR-9	ELAVL1	No	Inhibit pyroptosis	↓
CACR	miR-214-3p	/	No	Promote pyroptosis	↑
/	miR-21-3p	AR	Yes	Promote pyroptosis	↑
circRNA-010567	miR-141	/	Yes	Promote fibrosis	↑

*AMPK, AMP-activated protein kinase; MNS, 3,4-methylenedioxy-β-nitrostyrene; TXNIP, thioredoxin interacting protein; BMP-7, bone morphogenetic protein-7; MALAT1, metastasis associated lung adenocarcinoma transcript1; GAS5, growth arrest-specific transcript 5; AHR, aryl hydrocarbon receptor; Kcnq1ot1, KCNQ1 opposite strand/antisense transcript 1; foxo3a, Forkhead box o3; ARC, caspase recruitment domain; ELAVL1, ELAV-like protein 1; CACR, caspase-1-associated circRNA; AR, androgen receptor.*

## NLRP3 Regulation

As mentioned previously, the NLRP3 inflammasome comprises protein complexes, NLRP3, ASC, and pro-caspase-1. NLRP3, a sensor of PAMPs and DAMPs, consists of three domains, namely, PYD, nucleoside triphosphatase domain (NACHT), and leucine-rich repeat. NACHT, known as adenosine triphosphatase domain, is composed of helical domain 1, helical domain 2, winged helix domain, and nucleotide-binding domain (NBD) (Sharif et al., 2019). ASC contains CARD and PYD. Pro-caspase-1 is composed of p10, p20, and CARD (Luo et al., 2017). The sensor recruits ASC after detection of stimuli. Subsequently, the multimeric complex interacts with procaspase-1 and forms the inflammasome, which promotes procaspase-1 autocatalytic cleavage into caspase-1 (Malik and Kanneganti, 2017). The activation of the NLRP3 inflammasome includes two processes, namely, induction of transcription of NLRP3, predecessors of caspase-1 and IL-1β, and subsequent assembly of NLRP3 with pro-caspase-1 and ASC (Zeng et al., 2020). In high glucose, NF-κB showed a high expression in cardiomyocytes. The NF-κB inhibitor decreased the expression of NF-κB in mRNA and the levels of NLRP3 and caspase-1 (Luo et al., 2014). Thus, the transcriptional activation of NLRP3 can be controlled by NF-κB (He et al., 2016). The assembly can be activated by a plethora of stimuli, including ATP, pore-forming toxins, and particulate matter, which may explain the activation of the inflammasome in multiple cardiovascular diseases. Unlikely to be sensed directly by PRRs, these stimuli trigger a series of cellular signaling events, mitochondrial dysfunction, Ca^2+^ mobilization, and ROS production (Kelley et al., 2019; Yang et al., 2019b).

Fibrosis is the chronic aberrant accumulation of extracellular matrix components from fibroblasts and myofibroblasts derived from various sources, including epithelial to mesenchymal transition (EMT, a process wherein the epithelial cells change into mesenchymal cells and lose adhesion and migration ability) (Stone et al., 2016; Alyaseer et al., 2020). After the binding of TGF-β to its receptor complex, Smad2 and Smad3 are activated through CT phosphorylation and then together form a polymer along with Smad4 to transport into the nucleus, which consequently activates the transcription of EMT. Studies have well supported that TGF-β is among the mediators of fibrosis of in DbCM. According to studies, the NLRP3 inflammasome promotes fibrosis by upregulating TGF-β *via* mature IL-1. In addition, the NLRP3 protein promotes the TGF-β/Smad pathway in the absence of activation of NLRP3 inflammasome (Alyaseer et al., 2020). Che et al. indicated that the NLRP3 inflammasome may also induce fibrosis through the TGF-β/Smad pathway, in view of the increasing levels of the TGF-β and Smad phosphorylation under IL-1 exposure (Che et al., 2020). Hence, the clear links between NLRP3 inflammasome and fibrosis remain to be further studied.

The activation of the inflammasome also promotes apoptosis, which further exacerbates DbCM. Zhang et al. (2018) found that high-glucose-induced apoptosis relies on the inflammasome and IL-1β in cardiomyocytes, and preventing inflammasome formation can ameliorate myocardial injury. The NLRP3 inflammasome also plays a crucial role in metabolic disturbances mentioned above. Activated NLRP3 may be associated with the shift toward the aliphatic acid metabolism, which is detrimental to the heart (Sharma et al., 2018). In a nutshell, the NLRP3 inflammasome partly contributes to structural and functional alternations in the diabetic heart.

## Absent in Melanoma 2 Inflammasome Regulation

Besides the NLRP3 inflammasome, the AIM2 inflammasome has been demonstrated to be involved in the regulation of pyroptosis in DbCM. dsDNA released by pyroptotic cells subsequently activates AIM2, further aggravating the lesion tissue. Lugrin and Martinon (2018) found that activated AIM2 induces apoptotic caspase-8, resulting in caspase-1-mediated cell death in caspase-1-deficient mice *via* a process similar to apoptosis. A study showed that rats with DM exhibited high AIM2 protein and cardiac dysfunction. The inhibition of AIM2 reduces caspase-1, IL-1β, and collagen I and III of heart tissue. Of note, high levels of ROS can activate AIM2 (Wang X. et al., 2019). Previous studies have shown that prolonged ROS induced by hyperglycemia impairs mitochondrial function and decreases mitochondrial DNA copy number (mtDNA-CN) in retinal ECs (Al-Kafaji et al., 2016). The mtDNA-CN contains coding genes, which encode crucial proteins maintaining normal function of the mitochondria. The mtDNA-CN indicates the quantity of the mitochondria (Al-Kafaji et al., 2018). The mtDNA is released during mitochondrial stress caused by potentially dietary lipid overload or infection, leading to the formation of the AIM2 inflammasome (Sharma et al., 2019). The possibility is that ROS induced by hyperglycemia damage the mitochondria, subsequently inducing increased cytoplasmic mtDNA and finally activating AIM2. Oxidized low-density lipoprotein activates AIM2. However, compared with the NLRP3 inflammasome in DbCM, the pathways associated with the induction of myocardial fibrosis by AIM2 remain unknown. The association between TGF-β and AIM2 needs to be further investigated.

## NcRNA Regulation

Recently, ncRNAs have attracted considerable attention due to their ability to regulate gene expression. NcRNAs are translated from genome and regulate several cellular events *via* regulating the genetic network to affect protein effectors, including differentiation, cell metabolism, transcription, and proliferation (Wang J. et al., 2019). Increasing studies have exhibited the role of ncRNAs in the pathogenesis of pyroptosis in the diabetic heart, including microRNA (miRNA), circular RNA (circRNA), and long ncRNA (lncRNA) (Zhang et al., 2019).

MicroRNAs are endogenous ncRNAs with nearly 22 nt RNAs that regulate gene expression by targeting mRNAs for degradation or translational repression (Bartel, 2004). The research of miRNAs began in 1993, and now miRNAs serves as important therapeutic targets for several diseases (Almeida et al., 2011). Of note, about 90% of the total miRNAs in the human heart are constituted of 18 miRNA families and linked with cardiac diseases (Kumarswamy and Thum, 2013). Numerous miRNAs are involved in the process of DbCM, such as regulating cardiac hypertrophy, myocardial fibrosis, inflammation, and pyroptosis (Zhang et al., 2019). Remarkably, recent studies have revealed the KCNQ1 opposite strand/antisense transcript 1 (Kcnq1ot1) regulates pyroptosis in DbCM. MiRNAs associated with pyroptosis include miR-30d, miR-21-3p, and miR-9. Pan et al. (2013) found that increased circulating miR-30 can be used to diagnose cardiac hypertrophy. In high glucose, miR-30d is upregulated in cardiomyocytes with remarkably increased expression of caspase-1 and downregulation of Forkhead box O3 (foxo3a). Mir-30d directly targets Foxo3a, a crucial regulator of the cell cycle, oxidative clearance, and apoptosis. Elevated miR-30d in diabetic hearts suppresses foxo3a and caspase recruitment domain (ARC) expression and enhances the expression of inflammatory molecules to promote pyroptosis. As an miR-30d downstream protein, ARC was proven to protect cardiomyocytes against oxidative stress and doxorubicin cardiotoxicity (Zhang and Herman, 2006; An et al., 2009). Therefore, cardiomyocyte pyroptosis could be induced by miR–30d/foxo3a/ARC/caspase-1 pathway (Li et al., 2014). Androgen receptor (AR), mir-21-3p, NLRP3, and caspase-1 were elevated in high-glucose-treated cardiac fibroblasts. Furthermore, the expression of NLRP3 and caspase-1 decreased after silencing miR-21-3p. A previous study found that miR-21-3p can enhance caspase-1 and NLRP3 expression to aggravate pyroptosis of diabetic cardiac fibroblasts by suppressing AR (Shi et al., 2021). AR, a kind of steroid hormone receptor, plays a vital role in maintaining the structure and function of the cardiac tissue. ARs in myocardial tissues recognize androgens to regulate the cardiac phenotype and hypertrophy (Huang et al., 2016). Moreover, miR-9 plays a potential role in pyroptosis through targeting ELAVL1. The downregulation of miR-9 in diabetic heart corresponds with an increase of ELAVL1, caspase-1, and IL-1β, while miR-9 mimic transfection inhibits their expression (Jeyabal et al., 2016). These findings may validate the modulation of miR-9 in ELAVL1-dependent pyroptosis.

Long non-coding RNAs are widely defined as RNAs that are longer than 200 nt (Shi et al., 2013). CircRNAs are characterized by covalently closed loop structures without 3′ tails and 5′ caps (Chen and Yang, 2015). The competitive endogenous RNA (ceRNA) theory explains how lncRNAs and circRNA function as sponging miRNAs (Tay et al., 2014). Studies have reported the ceRNAs in the process of pyroptosis. Wu et al. (2021) found that increased metastasis-associated lung adenocarcinoma transcript 1 (MALAT1) enhances pyroptosis in high-glucose-induced cardiomyocytes *via* binding to miR-141-3p, which indicates that MALAT1 potentially has an impact in the pyroptosis of DbCM. Moreover, increased MALAT1 was found to enhance cardiac apoptosis in diabetic mice. The suppression of MALAT1 showed the opposite effects (Wang et al., 2021). Therefore, the knockdown of MELAT1 may effectively reduce myocardial injuries in DbCM. However, no distinctive evidence points out that MALAT1 mainly promotes pyroptosis or apoptosis in DbCM. With regard to the experiment using high-glucose-induced cardiomyocytes by Wu et al. (2021) further experiments on animal models are needed to learn more about the role of MALAT1 in DbCM. Kcnq1ot1, a kind of lncRNA, is present on human chromosome 11p15.5 (Kanduri, 2011). Kcnq1ot1 downregulates multiple genes located in the kcnq1ot1 domain *via* DNA-modifying proteins, and recruitment of chromatin. Kcnq1ot1 is closely associated with many heart disorders. Lai et al. (2020) indicated that Kcnq1ot1 regulates cardiomyocyte apoptosis in heart failure by targeting fusion in sarcoma. Li et al. (2017) elucidated that the down-regulation of Kcnq1ot1 inhibits myocardial ischemia reperfusion injury with acute myocardial infarction. In addition, emerging studies have proven that Kcnq1ot1 is closely related with pyroptosis in DbCM. Kcnq1ot1 is upregulated in the diabetic heart, which increases caspase-1 by targeting miR-214-3p. Silencing Kcnq1ot1 alleviates DNA fractionation and reduces calcium overload and hyperglycemia-treated cardiac fibroblasts (Yang et al., 2018a). Elevated caspase-1-associated circRNAs (CACR) in cardiomyocytes induced by high glucose also drive pyroptosis by targeting miR-214-3p. The knockdown of CACR can ameliorate caspase-1 activation. Meanwhile, CACR is also increased in the serum of patients with DM (Yang et al., 2019a), indicating that it can be used as a biomarker for DbCM. LncRNA growth arrest-specific transcript 5 (GAS5) is repressed in cardiomyocytes in DbCM. GAS5 is closely related to cardiovascular diseases. Wang et al. (2016) elucidated that GAS5 promotes hypertension-induced vascular remodeling. Moreover, Liu et al. (2019) found that the overexpression of GAS5 decreases cardiac fibrosis in mice *via* the PTEN/MMP-2 pathway. Xu et al. (2020) found that GAS5 is repressed in the cardiomyocytes of mice with DbCM, the upregulation of which enhances cardiac function and inhibits NLRP3 and caspase-1. Highly expressed GAS5 represses the expression of miR-34b-3p. Meanwhile elevated expression of miR-34b-3p induces the expression of aryl hydrocarbon receptor (AHR). Thus, this study suggested that GAS5 overexpression inhibits NLRP3-mediated pyroptosis *via* the miR-34b-3p/AHR pathway (Xu et al., 2020). As a negative regulator of NLRP3, AHR binds to the xenobiotic response element regions to block NF-κB to inhibit the expression of NLRP3 (Huai et al., 2014). Therefore, GAS5 and AHR may be vital targets for the treatment of DbCM.

The process of cardiac fibrosis is a vital event in DbCM. Tang et al., indicated that circRNA-000203 is over-expressed in the diabetic myocardium, which increases the expression of α-SMA, Col1a2, and Col3a1 in cardiac fibroblasts. Zhou and Yu (2017) suggested that another circRNA could promote myocardial fibrosis. They found that circRNA_010567 modulates miR-141 and the expression of TGF-β1, mediating fibrosis-associated protein resection (Zhou and Yu, 2017). However, compared with lncRNAs and miRNAs, we know less about the molecular mechanisms of circRNA in DbCM.

Taken together, the upregulation and downregulation of these ncRNAs in cardiomyocytes play an important role in the progression of DbCM. Moreover, the network of lncRNA/circRNA–miRNA–mRNA may provide insights into the therapeutic targets for DbCM (Li M. et al., 2019).

## Autophagy Regulation

Recent studies indicated the potential interplay between pyroptosis and other forms of death. Zhang et al. (2020) found that LC3-II/I is reduced and p62 is increased in the myocardium of diabetic rats, suggesting that autophagy is inhibited. Along with suppressive autophagy, increased NLRP3 inflammasome is detected. Further study demonstrated that rapamycin, an autophagy activator, enhances the level of LC3-II/I, decreases p62, and inhibits the activation of NLRP3 inflammasome to alleviate myocardium injury. In addition, autophagy can clear injured tissues and stress products such as ROS, which can induce NLRP3 inflammasome activation. Therefore, autophagy can inhibit NLRP3 inflammasome induced by high glucose, which can be a new target for DbCM treatment (Zhang et al., 2020). However, how high glucose suppresses autophagy and whether high glucose promotes cells undergoing autophagy to embark in pyroptosis remain unknown. Song et al. (2021) found that sirtuin3 (SIRT3) is inhibited in the myocardium of diabetic mice, which further triggers severe cardiac injury. The level of necroptotic-related proteins receptor-interacting protein kinase (RIPK) 1/3 is enhanced in cardiomyocytes of SIRT3-knockout diabetic mice. Moreover, the expression of NLRP3 inflammasome in SIRT3-knockout diabetic mice is much higher than that in wild-type diabetic mice (Song et al., 2021). Furthermore, previous studies confirmed that PIPK3 participates in inflammasome activation and cytokine release. The PIPK1-PIPK3 cell death platform called necrosome forms after some related receptors are activated. Then, PIPK3 phosphorylates its downstream enzyme mixed lineage kinase domain-like protein, which combines to membrane and forms membrane pores. This results in the release of cytoplasmic contents and DAMPs, further triggering NLRP3 inflammasome (Frank and Vince, 2019). Evidence proves that ROS can activate necroptosis, which indicates the promotional function of pyroptosis to necroptosis in DbCM.

## Drug Regulation

Pyroptosis is detrimental to the heart and speeds up the progression of DbCM. In this review, we summarized several treatment ideas to alleviate DbCM by hindering pyroptosis.

First, initial glycemia and metabolic disorders induce pyroptosis in cardiomyocytes, which may be prevented by early use of hypoglycemia drugs to protect cardiomyocytes. For example, previous studies have reported that metformin, an antidiabetic drug, could play a cardioprotective role. Studies have shown that metformin can upregulate the phosphorylation of AMP-activated protein kinase (AMPK), which develops glycolysis to maintain cellular energy balance (Choi and Park, 2013; Driver et al., 2018). In addition, Yang et al. (2019a) implicated that metformin represses the NLRP3 inflammasome *via* the AMPK/mTOR pathway in DbCM. Empagliflozin highly selectively inhibits sodium glucose cotransporter-2. Xue et al. (2019) suggested that empagliflozin inhibits cardiomyopathy through the SGC-CGMP-PKG pathway in mice with type 2 diabetes. Clinical trials have shown the efficiency of empagliflozin in patients with type 2 diabetes (Frampton, 2018; Xue et al., 2019).

Second, the inhibition of inflammasomes offers potential therapeutic targets for DbCM, including transcriptional suppression of inflammasome components, assembling prevention and blockade of active caspase-1 and GSDMD cleavage (Zeng et al., 2019). The NF-κB inhibitor BAY-11-7082 decreases the expression of inflammasome components in cardiomyocytes treated with high glucose. On the basis of previous reports, BAY-11-7082 exhibits strong anti-inflammatory effects by inhibiting κB kinase of NLRP3 priming process and reducing the translocation of NF-κB subunit (Lee et al., 2012). Regarded as the second messenger in the activation of NLRP3, ROS may affect NF-κB signaling and later assembly process. In cardiomyocytes treated with high glucose, increased ROS can promote thioredoxin interacting protein, which binds NLRP3 inflammasome directly and promotes its assembly (Luo et al., 2014). Studies have found that the ROS scavenger Eukarion (EUK-134) can block inflammasome dissociating TXNIP and NLRP3 inflammasome (Sho and Xu, 2019). Skimmin has recently been found to play a significant role in DbCM. It can reduce blood glucose and improve fibrosis in diabetic rats. Of note, skimmin enhances autophagy, which diminishes ROS generation and inflammatory reaction in a diabetic heart with decreased pyroptosis-related cytokines (Liang et al., 2021). Skimmin could serve as a promising protective medicine in DbCM. Previously, EUK134 has been synthesized and tested n heart protection as a mitochondria-targeted antioxidant (Ajith and Jayakumar, 2014). In this way, mitochondrial-targeted agents have great potential for cardioprotection. Novel NLRP3 inhibitors were recently found to inhibit inflammasome assembly. ATP is indispensable in the role of NIMA (never in mitosis gene)-related kinase 7 (NEK7) in activating inflammasome. The cryo-EM structure of NLRP3 in humans can bind to NEK7, a ser/thr kinase. NEK7 is phosphorylated to make NLRP3 active conformation when ATP binds to the NBD domain (Neha et al., 2019). Thus, according to the process, inhibitors of the inflammasome are produced by blocking the interaction of NEK7 and NLRP3 and active conformation for copolymerization, which prevents pyroptosis (El-Sharkawy et al., 2020). Parthenolide and 3,4-methylenedioxy-β-nitrostyrene suppress the ATPase of NLRP3 and thus inhibit the interaction of NEK7 and NLRP3. Caspase-1 inhibitors have also been researched widely. VX765 was demonstrated to inhibit the expression of GSDMD and IL-1β in the brain of mice (Xu et al., 2019). However, further experiments are required to determine the effects of caspase-1 inhibitors such as VX765 in cardiomyocytes. Necrosulfonamide can bind to GSDMD directly and suppress membrane pore formation and IL-1β release in murine monocytes/macrophages, which provides ideas for the treatment of DbCM (Rathkey et al., 2018). Exogenous bone morphogenetic protein-7 was proved to inhibit NLRP3-related cell deaths and inflammatory response in a diabetic heart. Moreover, it improved heart remodeling, which makes it useful in several heart diseases related to adverse heart remodeling (Elmadbouh and Singla, 2021).

Third, the upregulation and downregulation of related ncRNAs also serve as a target. As AAV9-shNlrp3 alleviates atrial fibrillation, specific shRNAs may exert an influence on DbCM amid silencing the gene of caspase-1, NLRP3, and ASC (Zeng et al., 2019).

Fourth, the inhibition of AIM2 inflammasome provides a novel target. Connection with dsDNA is an essential factor for activation of AIM2 inflammasome. Thus, the molecules inhibiting the recognition of dsDNA and HIN domain to restrain AIM2 could be a therapeutic target of DbCM. Having a similar structure with mouse p202 human IF16-designated IFI16-β lacks a PYD but includes two HIN domains. P202 and IFI16 have higher affinity to connect dsDNA than AIM2 and prevents AIM2 oligomerization and ASC clustering by binding to HIN of AIM2 (Wang et al., 2018). CARD-only proteins and PYD-only proteins have been demonstrated to negatively regulate AIM2, which provides new ideas for the therapy of inflammatory disease (Indramohan et al., 2018). One tegument protein pUL83 released by human cytomegalovirus connects human AIM2 to attenuate AIM2 inflammasome activation (Huang et al., 2017). Likewise, VP22 released by herpes simplex virus-1 has been shown to inhibit human AIM2 (Maruzuru et al., 2018; Table 1).

## Conclusion and Outlook

Diabetic cardiomyopathy is a prevalent disease worldwide causing heart failure in patients with diabetes. Pyroptosis features the formation of membrane pore and leakage of proinflammatory intracellular contents such as IL-1β and IL-18, which is mainly activated by caspase-1 signaling pathways. Previous and emerging studies have shown that pyroptosis could be triggered by the canonical pathway with the involvement of caspase-1 and non-canonical pathway induced by caspase-11/4/5. In canonical inflammasome pathway, NLRP1/3, NLRC4, and AIM2 are triggered by sensing variety of DAMPs and subsequently collecting ASC, which recruits pro-caspase-1. In the non-canonical pathway, related caspase family members directly recognize LPS without the involvement of inflammasome assembly. Several factors including abnormal nutrient metabolism such as glucose and lipid; production of toxic metabolic waste products; and imbalanced electrolytes such as Ca^2+^ and cell death such as apoptosis and necrosis contribute to DbCM. Evidence has supported that pyroptosis is involved in the pathogenesis of DbCM. Related studies found the interplay between pyroptosis and other kinds of cell death such as autophagy and necroptosis. Activated autophagy inhibits NLRP3 inflammasome activation to repress pyroptosis. Moreover, necroptosis and pyroptosis could promote each other. In our review, we summarized the regulations of pyroptosis in DbCM, including NLRP3 inflammasome, lncRNAs, miRNAs, circRNAs, and AIM2 inflammasome. As a molecular marker, NLRP3 inflammasome plays a significant role in DbCM. NF-κB modulates NLRP3, and mitochondrial dysfunction and overproduction of ROS are also involved in the activation of NLPR3 inflammation activation. Various miRNAs, circRNAs, and lcnRNAs such as MALAT1, Kcnq1ot1, and GAS5 have been proved to regulate pyroptosis in DbCM *via* different pathways. Given the regulatory mechanisms that participate in pyroptosis, hypoglycemic drugs and gene targeting may be new treatment in the future. However, the role of pyroptosis in DbCM is still not very clear. In accordance with numerous studies, there are closed crosslinks between pyroptosis and apoptosis. Apoptosis could serve as the second signal of NLRP3 activation under ER stress. Caspase-3 is also proved to participate in pyroptosis. Moreover, apoptosis does not release cytokines and apoptotic cells that send signals to attract macrophage. While pyroptosis enlarges inflammatory size, it is seen as a kind of dirty cell death way. Therefore, the characteristic molecule to distinguish pyroptosis and apoptosis may be gasdermin family members such as GSDMD and GSDME, which promote the formation of membrane pores to facilitate pyroptosis. And the point is, what can regulate caspase-3 to promote GSDME? However, no study has proved that caspase-3 induces GSDME in cardiomyocytes treated with high glucose. Does ROS play a role in increasing cytoplasmic mtDNA to activate AIM2 inflammasome by impairing mitochondria? Further studies are needed to determine the molecular mechanisms. Some viral proteins could bind to AIM2 to inhibit AIM2 inflammasome formation, which may alleviate pyroptosis in DbCM. Given this, related vaccinations would be used in the treatment of DbCM in the future.

## Author Contributions

YFL and YQL wrote this review. ZW and JM modified this review. All authors contributed to the article and approved it for publication.

## Conflict of Interest

The authors declare that the research was conducted in the absence of any commercial or financial relationships that could be construed as a potential conflict of interest.

## Publisher’s Note

All claims expressed in this article are solely those of the authors and do not necessarily represent those of their affiliated organizations, or those of the publisher, the editors and the reviewers. Any product that may be evaluated in this article, or claim that may be made by its manufacturer, is not guaranteed or endorsed by the publisher.
